# Determinants of response to a parent questionnaire about development and behaviour in 3 year olds: European multicentre study of congenital toxoplasmosis

**DOI:** 10.1186/1471-2431-5-21

**Published:** 2005-07-05

**Authors:** A Salt, K Freeman, A Prusa, N Ferret, W Buffolano, G Malm, D Schmidt, HK Tan, RE Gilbert, The European Multicentre Study on Congenital Toxoplasmosis (EMSCOT)

**Affiliations:** 1 The Neurodisability Service, Great Ormond Street Hospital for Children and Institute of Child Health, London, UK; 2 Albert Einstein College of Medicine, Department of Epidemiology and Population Health, New York, U.S.A; 3 Department of Pediatrics, Division of Neonatology and Intensive Care, Medical University of Vienna, Austria; 4 CHU de NICE, Service Parasitologie – Mycologie, Hopital L'Archet II, BP 3079, 06202 NICE Cedex 3; 5 Perinatal Infection Unit, Dept of Pediatrics, University of Naples Federico II, Naples, Italy; 6 Karolinska University Hospital, Huddinge, Stockholm, Sweden; 7 Department of Parasitology, Staten Seruminstitut, Copenhagen, Denmark; 8 Centre for Paediatric Epidemiology and Biostatistics, Institute of Child Health, London, UK

## Abstract

**Background:**

We aimed to determine how response to a parent-completed postal questionnaire measuring development, behaviour, impairment, and parental concerns and anxiety, varies in different European centres.

**Methods:**

Prospective cohort study of 3 year old children, with and without congenital toxoplasmosis, who were identified by prenatal or neonatal screening for toxoplasmosis in 11 centres in 7 countries. Parents were mailed a questionnaire that comprised all or part of existing validated tools. We determined the effect of characteristics of the centre and child on response, age at questionnaire completion, and response to child drawing tasks.

**Results:**

The questionnaire took 21 minutes to complete on average. 67% (714/1058) of parents responded. Few parents (60/1058) refused to participate. The strongest determinants of response were the score for organisational attributes of the study centre (such as direct involvement in follow up and access to an address register), and infection with congenital toxoplasmosis. Age at completion was associated with study centre, presence of neurological abnormalities in early infancy, and duration of prenatal treatment. Completion rates for individual questions exceeded 92% except for child completed drawings of a man (70%), which were completed more by girls, older children, and in certain centres.

**Conclusion:**

Differences in response across European centres were predominantly related to the organisation of follow up and access to correct addresses. The questionnaire was acceptable in all six countries and offers a low cost tool for assessing development, behaviour, and parental concerns and anxiety, in multinational studies.

## Background

Measurement of children's development, behaviour, and impairment is essential in studies that seek to determine the impact of early life events on functional abilities. Because professional administered standardised assessments are extremely resource intensive, parent-completed questionnaires are used increasingly, particularly in large studies of populations at low risk of impairment [1-4]. Uncertainties about the validity of parent-reported outcomes have been addressed by several studies showing that, compared with professional assessments, parents correctly report moderate to severe cognitive or speech and language impairment, behavioral problems, and disability [3,5-12]. Much less is known about the reliability and acceptability of parent-completed questionnaires in different countries, languages and cultures, except for tools measuring behaviour or quality of life [13-17]. Such information is particularly relevant for multinational studies.

This report is based on a prospective multicenter cohort study, The European Multicentre Study on Congenital Toxoplasmosis (EMSCOT), which was initiated to determine the effects of congenital toxoplasmosis and prenatal treatment on development, behaviour, specific impairments, and parental anxiety. We were constrained by the need for the assessment tool to be low cost, require minimal input by local investigators, cover all domains of development, and avoid measurement of vocabulary or other language-specific attributes. In addition, we wanted a tool that maximised response, minimised bias among responders, measured the same entity, and was similarly acceptable, in all six countries studied. The aim of the tool was to detect moderate to severe abnormality in the outcomes measured.

In order to assess the potential for bias when using the postal questionnaire, we examined the influence of organizational factors within centers and characteristics of the individual child, on three outcomes: response to the questionnaire, the age at response, and, among responders, completion of the drawing tasks by children. The aim was to determine the applicability and acceptability of this low cost tool in different European settings.

## Methods

### Study population

**Figure 1:**
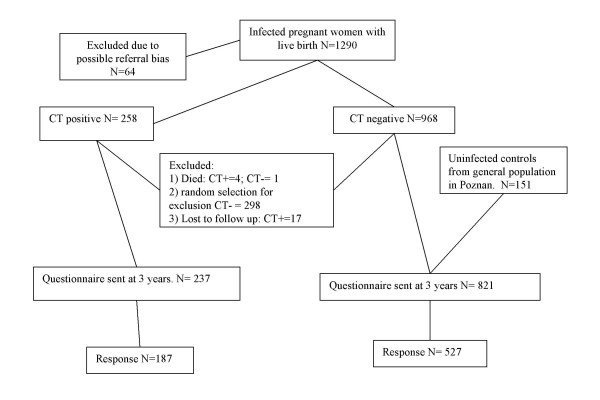
**Flow diagram to show recruitment into the study**. Flow diagram to show recruitment into the study. Criteria for possible referrals reported elsewhere [18]. CT = congenital infection status: - depicts uninfected, and + infected children.

The study population comprised children with and without congenital toxoplasmosis born to women exposed to toxoplasmosis in pregnancy (Figure 1). In eight centres (six in France, one in Vienna, and one in Naples), women were identified by prenatal screening. In Stockholm, infected women were identified by retrospective testing of stored prenatal samples, and in two centres (Copenhagen and Poznan), children were identified by neonatal screening for congenital toxoplasmosis. We enrolled all children in the French centres, where the ratio of uninfected to infected was about three to one. However, in Vienna, Stockholm and Naples there were about nine uninfected children to each infected child. We therefore randomly selected four uninfected children for each infected child for inclusion in the survey at three years of age. In Poznan and Copenhagen, only infected children were identified by neonatal screening. Therefore in Poznan we enrolled the next six uninfected children born after each infected child who underwent Guthrie Card screening. No uninfected children were enrolled in Copenhagen. More than 90% of women received prenatal anti-toxoplasma treatment in France, Vienna, and Naples. In the other centers, none of the women were treated. The screening schedule and duration of postnatal treatment of infected children are summarized in Table 1 and reported in more detail elsewhere [18].

**Table 1:** Summary of clinical management and follow up protocols, and response rates for each center

				Organizational attributes				
**Study Center**	Total sent questionnaires	Prenatal re-testing interval (months)^1^	Duration post-natal treatment (month)	A	B1	B2.	C	Total
**FRANCE**								
Lyon^1^	184	1	14	3	0	3	3	9
Paris^1^	182	1	12	1	1	2	2	6
Grenoble^1^	34	1	12–24	1	0	1	1	3
Marseille^1^	91	1	12–24	3	1	3	3	10
Nice^1^	44	1	24	4	0	3	3	10
Toulouse^1^	73	1	12	4	0	2	2	8
**AUSTRIA**								
Vienna^1^	187	3	12	4	1	3	2	10
**ITALY**								
Naples^1^	53	3	12	4	1	3	3	11
**SWEDEN**								
Stockholm^1^	16	NS^4^	12	4	1	2	1	8
**POLAND**								
Poznan^2^	180	NS	12	4	1	3	2	10
**DENMARK**								
Copenhagen^3^	14	NS	3	1	1	1	2	5
**TOTAL**								

NS = neonatal screening

Uninfected children: ^1 ^born to infected women; ^2 ^sampled from general population, ^3 ^none ^4 ^Detection of maternal infection based on neonatal testing of neonatal Guthrie card bloodspots and retrospective testing of stored maternal serum

Organizational attributes

A. Degree of local study clinician direct involvement in follow-up (FU) of children and contact with child's local paediatrician

4 = FU >= 75% children and regular contact

3 = FU >= 50% children and regular contact

2 = FU < 50% children and some contact

1 = FU <25% and no regular contact

B. Access to addresses

B1: 1 = National or local address register

0 = No use of population address register

B2: 3 = regular contact with parents/paediatricians to update addresses >50%

2 = initial contact address >50% but additional methods to update addresses

1 = >75% through initial contact address only

C. Direct contact with parents to encourage return of questionnaire

3. Telephone contact and special letter

2. Follow-up letter

1. No special efforts

### Postnatal follow up

All children born to toxoplasma infected women had paediatric, ophthalmic and cranial ultrasound examinations in early infancy, and infected children were assessed annually [18]. The exception to this rule was the group of uninfected children in Poznan who were not offered specialist clinical follow up. At 36 months of age, a questionnaire was mailed to parents together with a stamped addressed reply envelope, an information sheet, and crayons for the child. Two reminders were mailed to non-responders at 2 monthly intervals.

After the study, we sent a questionnaire to each centre to measure organisational attributes, such as whether local study investigators were directly involved in provided clinical follow up for the child, whether they had regular contact with the child's own paediatrician, access to a central address register for tracing families, and contact with families to encourage response. These factors were summed to generate a total unweighted score (see Table 1).

### Outcomes

The questionnaire consisted of 30 questions measuring motor, speech and language, and cognitive development, behavior, parental concerns about development, parental anxiety about the health of their child now and in the future, referral to a specialist, and specific impairments (including vision, hearing, cerebral palsy, and epilepsy). The full version is available at: . The assessment tools from which the questions were derived are summarized in Table 3. For behavior, we used the entire assessment tool, as validated in a large community sample of children [12]. However, for speech and language, and cognition, we scrutinized the correlation coefficients in unpublished data provided by the developers of these tools and, with their permission, selected those items which were most predictive and independent. We considered that the population of children born to toxoplasma infected women would be similar to the general community populations in which these assessment tools had been validated. Although components of the questionnaire have been validated, the entire questionnaire in the format used in this study has not yet been validated.

**Table 3:** Source for questions measuring development, behavior, and parental concerns and anxiety

**Outcome**	Question number in questionnaire*	**Score range**	**Source**
**Development and behavior**			
Motor development	6 (a–h)	0–16	Griffiths Mental Development Scales [23], Denver Developmental screening test [24]
Speech Development Language development	10 (a–c) 10 (d–g)	0–6 0–8	General Language Screen**, parent completed questionnaire for 3 year olds [25]. Validated against four standardised speech and language tests administered by an assessor.
Cognitive ability (non-verbal)	11 (a–g)	0–7	PARCA3** (Parent Report of Children's Abilities) [6] validated for 3 year olds against the MacCarthy Scale
Behavior	13 (a–y)	1–16	'Strengths and difficulties questionnaire' (SDQ), validated in 3 to 16 year olds against clinician assessment of behavior disorder [10,12]. Entire questionnaire, published translations, and scoring algorithm used.
**Parental concerns, specialist referral, and parental anxiety**			
Parental concerns a) Learning, behavior, development b) Speech and language	5 8	1–3 1–3	Adapted from PEDS** (Parent Evaluation of Developmental Status). Predicts risk for developmental and behavioral problems and the need for clinical assessment.[26,27]
Impact of behavior on family	14, 14a–d	0–2	SDQ questionnaire [10,12,28]
Parental anxiety	25,26,27	0–15	Adaptation of rating scales for measuring anxiety during pregnancy and postpartum [29] in relation to antenatal screening (numbered six point horizontal scale with verbal anchors at extremes).
**Child completed questions**			
Cognitive and fine motor skills Copying a line, circle, and cross. Draw a man	12 (a–c) 30	0–3 1–24	The child's ability to copy a circle, line and cross was assessed using scoring and normative data available from the Beery Buktenica Developmental Test of Visual Motor Integration [30]. The 'draw a man' was scored using a standardised system and normative data from the Goodenough Draw a Man test [31], using raw scores.
**Confounding variables**			
Education level achieved	28	0–3	Educational level achieved based on standard categories defined by the Organisation for Economic Co-operation and Development (OECD) for Europe.[32]

* Full version of questionnaire available on

**A subset of the most predictive and least correlated questions were selected.

### Questionnaire development and piloting

Development of the questionnaire involved collaborating pediatricians, obstetricians, parasitologists and psychologists in different countries to ensure that questions would be widely understood and acceptable. Questionnaires were translated into the six languages in the study, back translated to English by someone unaware of the original English version, and compared to the original version to ensure meaning was retained. The questionnaire was piloted in the six countries in general pediatric outpatient clinics, high risk (preterm) follow-up clinics, and day care centers, and parents were asked about difficult or offensive questions, the length of the questionnaire, and how long it took to complete. Research ethics approval was obtained for all participating centres

### Analyses

#### Development of scores

To summarize the responses relating to development and parental anxiety, unweighted scores were derived without knowledge of infection or treatment status. Spearman correlation coefficients were used to identify redundancies among items, and the final scale was based on items with relatively low inter-item correlations. If less than 50% of answers were missing for each outcome, the total score was prorated. For behaviour, and the children's drawings, we used the published scoring systems (see Table 3). All scores were coded so that a high score was abnormal. One third of the children's drawings were scored by a second assessor and discrepancies reviewed.

#### Analysis of response

We developed multivariate models to identify factors associated with each of the three outcomes: a) whether the questionnaire was completed and returned; b) the child's age at questionnaire completion; and c) whether the child completed the 'draw a man' task. Age at response was analysed as a surrogate marker for 'response or not' that might be susceptible to family as well as center factors. The child-completed task was included to assess its acceptability and the potential bias involved in such assessments that require additional effort from the family.

Initially, we examined the heterogeneity of effects within French centres, and found significant differences in response across centres. Thus, we decided to use a hierarchical generalized linear model for dichotomous outcomes (response to the survey, and response to 'Draw a Man') to account for heterogeneity among centers within France, along with other centres in the model. A generalized estimating equation (SAS Version 9.1 PROC GENMOD with the ASSESS options to assess fit of the model) with centers nested within country was derived to determine characteristics associated with response to questionnaire, and completion of the 'draw a man' task. Goodness of fit was assessed using the Pearson Chi-square result divided by its degrees of freedom. Values close to 1 indicated lack of overdispersion of the model [19]. To predict child's age at survey completion, we used multiple linear regression. Lyon, the largest centre, was used as the reference category.

The models examined the effect of centre, and the score for centre organisational attributes. We also examined the effect of the patient characteristics (see Table 4), including the presence of intracranial lesions, or abnormal neurological findings (microphthalmia, microcephaly, seizures, or abnormal neurological examination requiring referral to a specialist) before 4 months of age. This cut-off was chosen as the number of examinations was similar for infected and uninfected children up until this age.

**Table 4:** Characteristics associated with response to questionnaire (N = 1058 total)

Characteristic	Number responding (%)	Odds ratio for response^4 ^(95% confidence interval)	Final model: Adjusted odds ratio^5 ^(95% CI)
All centers	714 (67.5)		
**Center Variables**			
Lyon (reference)	134 (72.8)	reference	
Paris	91 (50.0)	0.37 (0.24, 0.57)	
Grenoble	8 (23.5)	0.11 (0.05, 0.27)	
Marseille	69 (75.8)	1.16 (0.65, 2.06)	
Nice	33 (75.0)	1.12 (0.53, 2.38)	
Toulouse	44 (60.3)	0.56 (0.31, 0.99)	
Copenhagen	9 (64.3)	0.67 (0.21, 2.1)	
Vienna	134 (71.7)	0.97 (0.61, 1.54)	
Stockholm	8 (50.0)	0.37 (0.13, 1.05)	
Naples	50 (94.3)	6.22 (1.85, 20.84)	
Poznan	134 (74.4)	1.09 (0.68, 1.73)	
**Total score for organisational attributes^3^**	9 (6,10)	1.36 (1.27, 1.45)	1.15 (1.09, 1.23)
**Infection status^1^**			
CT+	178 (80%)	2.95 (2.01, 4.31)	4.96 (3.58, 6.88)
CT-	527 (64%)		
**Maternal age^1,2,3 ^***(mean years, 95% CI)*	582 (67%) *28.4 (27.6, 29.2)*	1.03 (0.99, 1.07)	1.02 (0.99, 1.05)
**Parity^1,2,3 ^***(mean, 95% CI)*	503 (67%) *0.9 (0.8, 1.0)*	1.05 (0.90, 1.23)	
**Gestational age at birth^1,2,3 ^***(mean weeks, 95% CI)*	530 (68%) *39.0 (38.8, 39.1)*	1.00 (0.92, 1.09)	
**Child's gender^1,2^**			
Male	379 (68%)	Reference	
Female	316 (68%)	0.94 (0.71, 1.25)	
**Prenatal treatment^1,2^**			
Any prescribed	532 (67%)	1.85 (0.98, 3.49)	
None	62 (66%)		
**Neurological abnormality and/or intracranial lesions^1,2^**			
Yes	25 (83.3)	2.69 (0.56, 13.00)	
No	569 (66.3)		

^1 ^Excludes 14 infected children from Denmark (9 responded), as no uninfected controls available.

^2 ^Excludes 156 children in Poznan (134 responded), as uninfected control group selected from the general population had no information on these variables.

^3 ^Odds ratio per unit increase in characteristic

^4 ^Adjusted for congenital infection status and center

^5 ^Adjusted for all factors shown. Goodness of fit statistic was close to 1 (1.0063)

As the total score for organizational attributes (given in Table 1) was a proxy for centre, we repeated all analyses, initially adjusting for centre, and then adjusting for the total unweighted score for organizational attributes. Potential covariates were added to a model with congenital infection status and center to determine the magnitude of association with outcome. Variables with associations that resulted in p-values less than .20 were included in the initial multivariate model. A monitored backwards stepwise approach was conducted, and models were assessed for convergence. The final model included only variables (or categories of variables) significant at p < .05. Bivariate associations were assessed using a Chi-square or Exact test for categorical characteristics and Wilcoxon Rank Sum tests for ordinal or non-normally distributed characteristics. The best fitting, and most parsimonious model was included in the results presented. Odds ratios or estimated means are presented along with 95% confidence intervals.

## Results

### Piloting of questionnaire

115 parents completed pilot questionnaires (70 healthy, 31 seen in pediatric clinics for clinical problems, 14 not specified), in France (32), Italy (40), Sweden (9), Denmark (10), and Poland (24). On average, parents took 21 minutes to complete the questionnaire (range 6 to 40 minutes, SD 7.88). Most parents (88%) thought that the questionnaire was the right length, 5% reported it was short, and 7% too long. Sixteen (14%) had difficulty understanding, or objected to one or more questions, of which the most frequent were: 'maternal age when last in full time education', questions about behavior (three parents felt the questions were not suitable for the age group), and one cognitive question about puzzles which was removed.

### Survey of organizational attributes and reasons for non-response

Table 1 shows that Naples, followed by Nice, Marseille, Vienna, Poznan, and Lyon had the highest total scores for the level of direct involvement in follow up by the local study centre, access to addresses, and methods to encourage compliance with the postal questionnaire. These centres also had the highest response rates (see Table 2). Overall 67% (714/1058) of parents responded, but the rate ranged from 24% (8/34) in Grenoble to 94% (50/53) in Naples. Table 2 shows that the main reason given by the local study coordinator for non-response was lack of a correct address (accounting for 44%, 151/344 of non-responders). Few parents refused to participate (n = 60, 17% of non-responders). As no reason was given for most non-responders (37%; 129/344), we could not tell whether these parents had not received a questionnaire, had refused to participate, or had completed but failed to return their questionnaire.

**Table 2:** Summary of response rates for each center

			**Reasons for non-response (% non-responders)**			
**Study Center**	Response rate (%)	Total non-responders	Address not known	Refused to participate	No response	Other^1^
**FRANCE**						
Lyon	73%	50	12 (24)	1 (2)	37 (74)	0
Paris	50%	91	46 (50)	0	45 (49)	0
Grenoble	24%	26	1 (4)	0	25 (96)	0
Marseille	76%	22	17 (77)	0	4 (18)	1 (4)
Nice	75%	11	8 (73)	3 (6)	0	0
Toulouse	60%	29	21 (72)	0	8 (28)	0
**AUSTRIA**						
Vienna	72%	53	21 (40)	32 (60)	0	0
**ITALY**						
Naples	94%	3	1(33)	2 (66)	0	0
**SWEDEN**						
Stockholm	50%	8	3 (37)	1 (12)	2 (25)	2 (25)*
**POLAND**						
Poznan	74%	46	20 (43)	21 (46)	4 (9)	1 (2)
**DENMARK**						
Copenhagen	64%	5	1 (20)	0	4 (80)	0
**TOTAL**		344	151 (44)	60 (17)	129 (37)	4 (1)

^1 ^Other reasons eg. died, mentally retarded parents

### Analysis of determinants of response to questionnaire

As shown in Table 4, there were statistically significant differences between centres in the proportion of parents responding to the questionnaire. Response was more common in Naples, and less common in Paris, Grenoble and Toulouse, than in Lyon. A more parsimonious model involved replacement of the centre variable with the centre score for organisational attributes which was significantly associated with increased response. The only other significant factor in this model was congenital infection status. (see Table 4).

### Determinants of age at response

On average the questionnaire was completed at 39.7 months of age (95% CI: 39.5, 40.0; range 35.4 to 63.9 months). In the multivariable analysis, factors significantly associated with older age at completion were study centre (delayed in Paris, Vienna and Naples), duration of prenatal treatment and detection of a neurological abnormality and/ or intracranial lesions in the first 4 months of life (mean difference in months at response was 1.67; with standard error = 0.72; R^2 ^= 0.16.).

### Determinants of child's response to 'draw a man'

94% of children copied drawings of a line, circle, and cross, but only 70% of children responded to the request to 'draw a man.' In multivariable analyses, completion of 'draw a man' was more common in girls (OR 0.62; 95% CI 0.44, 0.88), age at completion of the questionnaire (Odds ratio 1.13 per additional month of age; 95% CI 1.06–1.21), and centre (children in Poznan were more likely to respond than in Lyon; odds ratio 2.53, 95% CI 1.36, 4.68). The goodness of fit statistic was 1.0092.

### Parent completed questions

Most questions (>99%) were completed. Questions with the lowest rate of completion were on hearing loss (94%), vision (92%), and age when mother was last in full time education (92%).

## Discussion

The score for organizational attributes varied between study centers and was one of the main determinants of response to the questionnaire. Centers where study clinicians were directly involved in patient follow up, had access to a central address register, and directly contacted parents to encourage return of the questionnaire, had the highest response rates. There was no evidence that organizational attributes were associated with age at response, nor with whether the child drew a man. Congenital infection status was strongly associated with response to the survey, but only weakly associated with age at response, and was not significantly associated with whether the child completed the 'draw a man' task.

The response rate to this parent report survey suggests that a parent-completed postal questionnaire on development, behavior, and parental concerns and anxiety is acceptable across the six European countries studied. The high response rate in this study was achieved by clinicians without dedicated research coordinators in the local centres, although there were dedicated staff centrally. In some centres clinicians were laboratory-based and not directly involved in follow up of the child. The results of this study should therefore be widely applicable.

Our findings concur with those of a systematic review of methods for increasing response rates to postal questionnaires [20]; response was higher among parents for whom the study was of most interest (those with infected children), and response was improved by follow-up contact. Other important elements of survey design highlighted in the review by Edwards et al included keeping the questionnaire short, and mailing a second copy.

Non-response can introduce bias if non-responders differ from responders with respect to prognostic characteristics. In our analyses, we found no evidence that non-responders differed in terms of maternal age, parity, or prenatal treatment or with respect to prognostic factors associated with poor developmental outcome such as gestational age at birth or abnormal clinical manifestations in early infancy. Difficulties tracing the correct address probably favored inclusion of infected children in our study, but we found no evidence for a bias in favor or against inclusion of more severely affected children.

The age at response was largely determined by the centre, and was not significantly associated with the organizational attributes. Although questionnaires were intended to be mailed as soon as they arrived at the local centre, actual practice may have varied. The centre effect may therefore be explained by unmeasured centre characteristics such as availability of staff to mail questionnaires. The weak association between duration of prenatal treatment and increased age at response may be a chance finding. However, return of questionnaires was delayed from children with an intracranial lesion or neurological abnormality. This may reflect difficulties contacting such families. A similar finding was reported in a cohort study of children born preterm; families with severely neurologically affected children were most difficult to contact (a higher proportion of families of children with severe disability repeatedly failed to attend appointments, moved frequently or were adopted or fostered) [21].

Although completion of individual questions was high, certain questions fared less well, most notably when the child was asked to 'draw a man'. Low response may be because the task is quite difficult for three year olds, the lower age limit for this test, as completion did improve with age. As child-completed tasks can provide additional objective information about development, inclusion of more age-appropriate tasks may be valuable [6,7]. Failure of the 6% to 8% of parents to answer the questions on vision and hearing may reflect parental uncertainty or lack of confidence in reporting medical information whereas functional information was well reported. The question on 'maternal age when last in full time education' received most comments during the pilot phase, and was less popular than a related question about highest level of education achieved, which has been used most widely in Europe [22].

One of the main limitations of the study is that reasons for non-response were poorly documented. Consequently, refusals to participate may have been underestimated, and acceptability may have been overestimated. A further limitation is that study centre clinicians were surveyed about the organizational attributes of their centre after the study, when they were aware of how response rates differed among centers. This may have favored overestimation of the importance of organizational attributes for response.

## Conclusion

The parent completed questionnaire was acceptable in 11 centres in six European countries. Differences in response appeared to be related to organisation of follow up, and access to correct addresses. The questionnaire offers a low cost tool for assessing development, behaviour, and parental concerns and anxiety, in multinational studies.

## Competing interests

The author(s) declare that they have no competing interests.

## Authors' contributions

*Members of the European Multicentre Study on Congenital Toxoplasmosis (EMSCOT)

*Coordinating Committee: *M-H Bessieres, W Buffolano, H Dumon, R Gilbert, E Petersen (chairperson), A Pollak, P Thulliez, M Wallon.

*Writing Committee: *Freeman K, Salt A, Prusa A, Malm G, Ferret N, Buffolano W, Petersen E, Gilbert RE (coordinator).

*Centres contributing data *(number of patients contributed to this report): M Paul (134; University Medical Sciences, Poznan), A Prusa, M Hayde, A Pollak (134; University Children's Hospital, Vienna), M Wallon, F Peyron (134; Hôpital de la Croix Rousse, Lyon), S Romand, P Thulliez (91; Institut de Puericulture, Paris), J Franck, H Dumon, P Bastien, E Issert (69; Hôpital de la Timone, Marseille; CHU de Montpellier), W Buffolano (50; Universita di Napoli, Naples), M-H Bessieres (44; Hôpital de Rangueil, Toulouse), N Ferret, P Marty (33; Hôpital de l'Archet, Nice), H Pelloux, H Fricker-Hidalgo, C Bost-Bru (8; Centre Hospitalier Universitaire de Grenoble), G Malm, B Evengard (8; Huddinge Hospital, Stockholm), E Petersen (0; Statenseruminstitut, Copenhagen), C Chemla, (0; Hôpital Maison Blanche, Reims), E Semprini, V Savasi (0; Milan).

*Study design and coordination: *R Gilbert (Principal Investigator), L Gras, Hooi Kuan Tan, J Rickett, A Salt, L Valenti (Institute of Child Health, London)

*Statistical analysis: *Freeman K, Gras L (Institute of Child Health, London).

AS developed the questionnaires, participated in analyses and wrote the paper. KF did the statistical analyses and wrote the paper. RG had the idea for the study, designed the study, obtained the funding, developed the questionnaires, coordinated the study, and wrote the paper. All authors contributed to the design of the study and the writing of the paper. All authors read and approved the final manuscript.

## Pre-publication history

The pre-publication history for this paper can be accessed here:


